# Revitalizing the Epigenome of Adult Jaw Periosteal Cells: Enhancing Diversity in iPSC-Derived Mesenchymal Stem Cells (iMSCs)

**DOI:** 10.3390/cells14090627

**Published:** 2025-04-22

**Authors:** Felix Umrath, Valerie Wendt, Gilles Gasparoni, Yasser Narknava, Jörn Walter, Bernd Lethaus, Josefin Weber, Victor Carriel, Meltem Avci-Adali, Dorothea Alexander

**Affiliations:** 1 Department of Oral and Maxillofacial Surgery, University Hospital Tübingen, 72076 Tübingen, Germany; felix.umrath@med.uni-tuebingen.de (F.U.); valerie.wendt@freenet.de (V.W.); yasser.narknava@med.uni-tuebingen.de (Y.N.); bernd.lethaus@med.uni-tuebingen.de (B.L.); 2 Department of Orthopaedic Surgery, University Hospital Tübingen, 72072 Tübingen, Germany; 3 Department of Genetics and Epigenetics, Saarland University, 66123 Saarbrücken, Germany; gillesgasparoni@gmail.com (G.G.); j.walter@mx.uni-saarland.de (J.W.); 4 Department of Thoracic and Cardiovascular Surgery, University Hospital Tübingen, 72076 Tübingen, Germany; josefin.weber@med.uni-tuebingen.de (J.W.); meltem.avci-adali@med.uni-tuebingen.de (M.A.-A.); 5 Department of Histology, Tissue Engineering Group, Faculty of Medicine, University of Granada, 18016 Granada, Spain; vcarriel@go.ugr.es

**Keywords:** bone engineering, jaw periosteal cells, reprogramming, iPSC-derived mesenchymal stem cells, rejuvenation, epigenetics, DNA methylation clock, transcriptomics, teratoma formation

## Abstract

Induced pluripotent stem cells (iPSCs) are rapidly emerging as a transformative resource in regenerative medicine. In a previous study, our laboratory achieved a significant milestone by successfully reprograming jaw periosteal cells (JPCs) into iPSCs, which were then differentiated into iPSC-derived mesenchymal stem cells (iMSCs). Using an optimized protocol, we generated iMSCs with a remarkable osteogenic potential while exhibiting lower expression levels of the senescence markers p16 and p21 compared to the original JPCs. This study aimed to explore the epigenetic landscape by comparing the DNA methylation and transcription profiles of iMSCs with their JPC precursors, seeking to uncover key differences. Additionally, this analysis provided an opportunity for us to investigate the potential rejuvenation effects associated with cellular reprogramming. To assess the safety of the generated cells, we evaluated their ability to form teratomas through subcutaneous injection into immunodeficient mice. Our findings revealed that, while the methylation profile of iMSCs closely mirrored that of JPCs, distinct iMSC-specific methylation patterns were evident. Strikingly, the application of DNA methylation (DNAm) clocks for biological age estimation showed a dramatic reduction in DNAm age to approximately zero in iPSCs—a rejuvenation effect that persisted in the derived iMSCs. This profound reset in biological age, together with our transcriptome data, indicate that iMSCs could possess an enhanced regenerative potential compared to adult MSCs. Future in vivo studies should validate this hypothesis.

## 1. Introduction

Due to the demographic development of the world’s population, the increased average life expectancy leads to an increase in patients with chronic age-related disorders developing additional chronic illnesses [1]. A common model of aging is based on cellular senescence triggered by extrinsic or intrinsic factors, e.g., irradiation, chemotherapeutic agents, DNA damage, telomere shortening, increased p16^Ink4A^ expression, or the overexpression of oncogenes [2]. Although senescent cells cannot replicate, they remain metabolically active and secrete a myriad of pro-inflammatory factors, referred to as the senescence-associated secretory phenotype (SASP) [2]. SASP components induce the deterioration of the neighboring tissues and also provoke a senescence state in their immediate environment. The causal role of senescence in the aging process has been demonstrated by the transgenic *INK-ATTAC* mouse model, based on the clearance of p16^+^ senescent cells, resulting in delayed tissue dysfunction and extended health and lifespan [3].

For the development of regenerative therapies using mesenchymal stem cells (MSCs), the quantity, quality, and availability of autologous MSCs are major limiting factors, especially in elderly patients with an impaired health condition and intricate medication requirements [4,5,6]. The discovery of induced pluripotent stem cells (iPSCs) in 2006 has opened the door to a potentially limitless source of stem cells, offering a promising avenue for the advancement in regenerative medicine therapies [7]. For bone regeneration purposes, jaw periosteal cells (JPCs) can be easily isolated during routine surgical procedures and they possess a remarkable ability to regenerate bone. Due to their exposure to significant mechanical stress from the mastication process, these cells are somehow pre-primed, making them particularly well-suited for regenerative applications in oral and maxillofacial surgery. In a comparative study, Yoshimura and colleagues analyzed the yield, expansion, and multipotentiality of rat MSCs isolated from bone marrow, synovium, periosteum, adipose, and muscle [8]. Their study showed evidence that synovia MSCs possess the highest chondrogenic potential, and periosteal MSCs the highest osteogenic potential. However, the recovery of large jaw bone defects remains a significant challenge for surgeons in this field. Additionally, JPCs face limitations due to cell senescence occurring during prolonged in vitro culture. In this context, iPSCs offer a promising solution, providing an unlimited source for generating iPSC-derived mesenchymal stem cells (iMSCs).

Like adult MSCs, iMSCs can be used for a broad spectrum of clinical applications ranging from the treatment of immunological disorders like GvHD, Crohn’s disease, or multiple sclerosis, to regenerative therapies for the treatment of bone defects, osteoarthritis, ischemic stroke, and many more [9,10,11]. Nevertheless, the clinical application of iMSCs remains challenging, with several issues yet to be resolved. One such issue is the selection of the most appropriate cell type for iPSC generation, due to the possible recovery of epigenetic memory [12]. Additionally, there is a necessity for the standardization of iMSC production, given the multitude of differentiation protocols currently in existence, with the method of differentiation expected to influence iMSC functionality [13,14]. Moreover, the generation of autologous iMSCs is costly and time-consuming, which hinders individual treatments and, for some applications, necessitates the establishment of cell banks for HLA-matching [15,16]. Finally, the legal requirements for approval are extremely high, which means a significant financial investment and, consequently, a considerable risk [17].

On the other hand, it has been shown that iMSCs derived from adult MSCs show signs of rejuvenation compared to the originating cells [18,19,20]. This rejuvenation could be of great advantage for regenerative therapies as well as for the treatment of age-related diseases in general, as it is known that the stem cell potential of MSCs, especially the osteogenic potential, decreases with increasing age [21,22,23,24]. Accordingly, in rodents, evidence has been presented indicating that young MSCs offer a superior treatment option to older cells in the context of ischemic stroke [25]. Additionally, an overall attenuation of aging has been demonstrated by the administration of juvenile MSCs, whereas MSCs derived from aged donors have been observed to induce physical frailty in mice [26,27].

During reprogramming, somatic epigenetic markers are reset to the pluripotent ESC-like state, marking the starting point of epigenetic aging from which it progresses throughout the lifespan [28]. Epigenetic changes, including DNA methylation, histone modifications, and chromatin remodeling, are a hallmark of aging and contribute to genomic instability, ultimately leading to age-related changes in mRNA transcription [28]. However, epigenetic changes are only one of many hallmarks of aging, and their relationship to other hallmarks is not well-understood and is the subject of ongoing research. Kabacik et al. showed that epigenetic aging, as measured by DNA methylation, correlates with nutrient sensing, mitochondrial activity, and stem cell composition, but not with cellular senescence, telomere attrition, or genomic instability [29]. Consequently, further exploration is necessary in order to elucidate the implications of epigenetic rejuvenation on the therapeutic potential of iMSCs.

In previous works, we succeeded in reprogramming jaw periosteal cells (JPCs) and in further differentiating them into iMSCs, showing comparative or even slightly enhanced tri-lineage differentiation capacities compared to those of the primary JPCs, in the in vitro culture [30]. In the present study, our objective was to obtain more insights into the phenomenon of iMSC rejuvenation. Therefore, it is necessary that we determine the biological age of the generated iMSCs.

While critical telomere length is usually associated with aging, telomere length has been shown to have only a modest correlation with chronological age (r = 0.5), whereas cellular aging measured by DNA methylation (DNAm) has a much higher correlation for solid tissues (r = 0.99) [31]. Cytosine-5 methylation within CpG dinucleotides, also called DNA methylation, is a particular type of epigenetic control. A random sampling of 485,577 CpG sites found that 11% of them were significantly correlated with chronological age [32]. Taking this further, approximately 3 million of the 28 million CpG sites in the human genome correlate with chronological age. By analyzing publicly available DNA methylation datasets, Horvath succeeded in establishing a method to predict the estimated age of tissues and organs of individuals, the so-called epigenetic clock [33]. This approach is based on measuring the methylation at 353 CpG sites, whose methylation levels were highly correlated with chronological age in multiple tissues.

Methylation clocks provide a more accurate measure of biological age compared to chronological age, potentially reflecting an individual’s health status and aging process. The underlying biology of DNA methylation is complex, and methylation patterns can vary significantly between individuals due to environmental factors, lifestyle, and genetics, which may affect the accuracy of age predictions. Epigenetic clocks can be generated differently using single or multiple tissue types, and tissues from children or adults on the basis of larger or fewer sets of CpGs. Technological platforms exist to measure DNA methylation, including sequencing approaches, microarrays, and quantitative PCR. A wide range of studies provided the evidence of a synchronicity of the DNA methylation age across different tissues (with some exceptions) in the same body. Even tissues consisting of cells with very different proliferation/regeneration rates such as brain and blood yielded a similar epigenetic age [34].

However, epigenetic clocks exhibit a weak association with clinical measures of physiological dysregulation. To overcome this weakness, a phenotypic age estimator was constructed by Levine and co-authors, considering clinical characteristics and the chronological age and using DNA methylation levels in blood, resulting in the automatic selection of 513 CpGs. This age estimator outperforms the first generation of age estimators for predicting mortality, multi-morbidity, or health and lifespan [35]. Similar to the single tissue’s clocks, DNAm PhenoAge can lead to biased age predictions in children or other non-blood tissues.

Methylomics is a rapidly evolving field, especially with advances in next-generation sequencing. However, it is still unclear which methylation state and gene expression changes iMSCs undergo during differentiation from iPSCs, as well as their biological relevance. The present study aimed to compare the methylation and transcription profiles of iMSCs and their originating JPCs to identify potential differences. Furthermore, a methylation analysis was used to investigate the potential rejuvenation of the reprogrammed cells. Finally, we evaluated the safety of the generated cells by studying teratoma formation in mice.

## 2. Methods

### 2.1. Cell Culture

Jaw periosteal cells (JPCs) derived from three donors were included in this study in accordance with the local ethical committee (approval number 618/2017BO2, approval date: 26.03.2019) and after obtaining written informed consent. Jaw periosteal tissue was extracted during routine surgery and JPCs were isolated and expanded as previously reported [36,37]. JPCs and iMSCs were grown in hPL5-medium (DMEM/F12 (Gibco, Darmstadt, Germany) containing 5% human platelet lysate (hPL, Institute for Clinical and Experimental Transfusion Medicine Tübingen), 100 U/mL penicillin-streptomycin (Pen-Strep, Lonza, Basel, Switzerland), and 2.5 µg/mL amphotericin B (Biochrom, Berlin, Germany).

Induced pluripotent stem cells (iPSCs, ethical approval number 074/2016BO2, approval date: 17 May 2016) were cultured on vitronectin (VTN)-coated plates and maintained in Essential 8 medium (E8, Thermo Fisher Scientific Inc., Waltham, MA, USA) with daily medium changes and passaged every 4–6 days using 0.5 mM EDTA (Thermo Fisher Scientific Inc., Waltham, MA, USA) and 10 µM Y27632 ROCK inhibitor (Selleck Chemicals LLC, Houston, TX, USA).

### 2.2. Generation of Integration-Free iPSCs from JPCs Using srRNA

JPCs were reprogrammed to iPSCs as previously published [38]. Briefly, JPCs were incubated in hPL5 medium containing 0.2 µg/mL recombinant B18R protein (eBioscience, San Diego, CA, USA) prior to transfection with a self-replicating VEE-OKSM-GFP srRNA. From day 1 to 5, transfected cells were incubated with hPL5 medium containing 25% B18R conditioned medium (BcM) and 1 µg/mL puromycin (Invivogen, Toulouse, France) to select transfected cells. Furthermore, 250 µM histone deacetylase inhibitor sodium butyrate (NaB, Selleck Chemicals LLC, Houston, TX, USA) was added to the medium to enhance reprogramming efficiency. At day 7, the medium was changed to E8 medium (Thermo Fisher Scientific Inc., Waltham, MA, USA) containing 25% BcM. Single iPSC colonies were picked and transferred into VTN-coated wells containing E8 medium + 10 µM Y27632 ROCK inhibitor (Selleck Chemicals LLC, Houston, TX, USA) and maintained in E8 medium with daily medium changes.

### 2.3. Differentiation of iPSCs into iMSCs

For iMSC differentiation, iPSCs were grown in 6-well plates and detached using a cell scraper on day 0 of iMSC differentiation. Cell aggregates were transferred into VTN-coated T75 flasks containing 10 mL of hPL5 medium with 10 µM Y27632. Cells were now labeled as iMSCs passage 0 (P0). On day 1, medium was changed to hPL5 medium with 10 μM SB431542 (Selleck Chemicals LLC, Houston, TX, USA). Cells were cultured in P0 until day 10 with medium changes every other day. On day 10, cells were detached using TrypLE Express (Thermo Fisher Scientific Inc., Waltham, USA) and passed through a cell strainer. To determine the percentage of differentiated cells, CD105 expression was measured using flow cytometry. Therefore, 5 × 10^4^ cells were resuspended in 50 µL FACS-buffer (PBS + 0.1% BSA + 0.1% sodium azide) with 4% Gamunex (Grifols Deutschland GmbH, Frankfurt, Germany) and 5 µL of mouse anti-human CD105-APC antibody (BioLegend, San Diego, USA) was added. After 15 min incubation on ice, samples were washed twice and then resuspended in 200 µL FACS-buffer. CD105 expression was measured using a Guava EasyCyte 6HT-2L instrument (Merck Millipore, Billerica, MA, USA). Then, iPS cell exclusion and enrichment of CD105^+^ cells followed by the calculation of the CD105^+^ percentage and subsequent seeding of 1 × 10^6^ CD105^+^ cells into a T75 flask containing 10 mL of hPL5. In the following passages, cells were maintained in hPL5 medium with medium changes every 2–3 days and passaged after reaching a >80% confluency.

### 2.4. Flow Cytometric Analysis of JPCs, iPSCs, and iMSCs

The expression of pluripotency markers (SSEA-4, TRA-1-60, and TRA-1-80) and MSC markers (CD44, CD73, CD90, and CD105) was analyzed by flow cytometry. Cells were detached using TrypLE Express and 1 × 10^5^ cells per sample were incubated on ice for 15 min in 20 µL blocking buffer (PBS, 0.1% BSA, 0.1 mg/mL sodium azide (Sigma-Aldrich, St. Louis, MO, USA) and 10% Gamunex (human immune globulin solution, Talecris Biotherapeutics GmbH, Frankfurt, Germany)). Then, 50 µL FACS buffer (PBS, 0.1% BSA, 0.1 mg/mL sodium azide) as well as phycoerythrin (PE) and allophycocyanin (APC) conjugated antibodies (see Table 1) were added and incubated on ice for 20 min. After two washing steps with 200 µL FACS buffer, flow cytometry measurements were performed using the Guava EasyCyte 6HT-2L instrument (Merck Millipore, Billerica, MA, USA).

### 2.5. Osteogenic Differentiation

To stimulate osteogenic differentiation, iMSCs were cultivated in osteogenic medium (DMEM/F12 + 10% hPL, 1% Pen-Strep, 1% amphotericin B, 0.1 mM L-ascorbic acid 2-phosphate (Sigma-Aldrich, St. Louis, MO, USA), β-glycerophosphate (AppliChem, Darmstadt, Germany), and 4 µM dexamethasone (Sigma-Aldrich, St. Louis, MO, USA)) with medium changes every other day. After 15–25 days, cells were fixed with 4% formalin and monolayers were stained with 1 mL of Alizarin red solution (40 mM and pH 4.2) for 20 min. Unbound dye was washed off with distilled water and images were taken using an inverted microscope (Leica, Wetzlar, Germany). Quantification of calcium phosphate precipitates stained with Alizarin red was performed as previously reported [39]. Briefly, bound Alizarin dye was solubilized with 10% acetic acid and the absorbance at 405 nm was quantified photometrically after neutralization with 10% NH_4_OH.

### 2.6. Gene Expression Analysis of JPCs and iMSCs

RNA isolation from JPCs and iMSCs was performed using the NucleoSpin RNA kit (Macherey-Nagel, Düren, Germany) following the manufacturer’s instructions. RNA concentration was measured using a Qubit 3.0 fluorometer and the corresponding RNA BR Assay Kit (Thermo Fisher Scientific Inc., Waltham, MA, USA). The cDNA synthesis was performed with 0.5 μg RNA using the SuperScript Vilo Kit (Thermo Fisher Scientific Inc., Waltham, MA, USA). The quantification of mRNA expression levels was performed using the real-time LightCycler System (Roche Diagnostics, Mannheim, Germany). For PCR reactions, commercial ALP, BGLAP, p16, and p21 primer kits (Search LC, Heidelberg, Germany), and DNA Master SYBR Green I (Roche, Basel, Switzerland) were used. The amplification was performed with a touchdown PCR protocol of 40 cycles (annealing temperature between 68–58 °C), following the manufacturer’s instructions. Copy numbers of each sample were calculated based on a standard curve (standard included in the primer kits), and normalized to the housekeeping gene glyceraldehyde-3-phosphate dehydrogenase (GAPDH). X-fold induction was calculated relative to the corresponding control samples.

### 2.7. Mouse Model for Teratoma Formation

For the animal experiments, 52 NOG mice (mouse strain NOD.Cg-Prkdc<scid>Il2rg<tm1Sug>/JicTac, 26 males and 26 females) were purchased from Taconic Biosciences (Lille Skensved, Denmark) and animal experiments were performed in accordance with the institutional animal welfare guidelines and after approval by the local committee of Animal Welfare in Tübingen (Regierungspräsidium Tübingen). JPCs, iPSCs, and iMSCs were derived from 3 human donors, and 5 animals were used per donor, so, in total, 15 animals per JPC/iPSC/iMSC group and 5 animals for the negative control (Matrigel without cells) were included in the animal study (Supplementary Figure S1).

The cells were detached using TrypLE Express (JPCs and iMSCs) or 0.5 mM EDTA (iPSCs). Thereafter, the cells were resuspended in PBS to a concentration of 20 × 10^6^ cells/mL and mixed with an equal volume of phenol red free Matrigel (8.4 mg/mL, determined by the Lowry method, lot number 3100002, Corning, NY, USA). The suspension was pulled up in syringes and kept on ice until injection. Then, 100 µL of the Matrigel/cell suspension (50 µL/50 µL) was injected subcutaneously into the flank of each mouse. Eight weeks after injection, the mice were sacrificed, and tumors, if present, were explanted and embedded in paraffin. Tumor diameters were measured and histological stainings were performed on the embedded samples.

Eight weeks after subcutaneous JPC/iPSC/iMSC injection, teratomas, mass formation, or small Matrigel samples were explanted. Harvested tissues were chemically fixed with 4% paraformaldehyde (PFA) and then washed with DPBS, dehydrated using an ascending ethanol series (50%, 70%, 80%, 90%, and 99%), and embedded in paraffin for sectioning. Using a microtome (MICROM GmbH, Walldorf, Germany), 5 μm sections were cut from paraffin-embedded tissues. Sections were placed on SuperFrost micro-scope slides (R. Langenbrinck GmbH, Emmendingen, Germany). Subsequently, the tissues were stained with hematoxylin–eosin (HE) for general histological evaluation, Alcian blue for the identification of acid proteoglycans [40,41], a modified Verhoeff staining for collagen and elastic fibers analysis (conventional Verhoeff staining was contrasted with picrosirius solution), and the Fontana–Masson picrosirius (FMPS) technique for melanin and collagen content simultaneous analysis [42]. Alizarin red histochemical method was used to evaluate mineralization.

### 2.8. DNA Methylation Profiling

For each sample, 750 ng of genomic DNA was used with the EZ-DNA methylation Gold kit (Zymo research, Freiburg, Germany) according to the manufacturer’s recommendations. To measure CpG methylation levels, 4 µL of converted DNA was used with the Infinium MethylationEPIC array v1 (Illumina, San Diego, CA, USA) according to the supplier’s protocol. Arrays were scanned on a Hi-Scan platform (Illumina). Raw data files were loaded and processed with the RnBeads software suite (v2.16) [43,44] using dasen normalization [45] and no background subtraction. Methylation levels were calculated as beta values ranging from 0 (=0% methylation) to 1 (100% methylation). All downstream analyses were performed in R. Enhancer sets annotations were derived from enhancerAtlas 2.0 (http://www.enhanceratlas.org/, accessed on 20 February 2025) and merged with the CpGs featured on the array. For the evaluation of epigenetic clocks, the methylclock package [46] was used.

### 2.9. Transcriptome Analysis

Then, 1 µg of total RNA, isolated using the RNA Mini kit (Macherey Nagel, Düren, Germany), was used for RNA sequencing. Quality control, read mapping, and counting were performed using the Nextflow-based nf-core/rnaseq pipeline (version 1.4.2, https://nf-co.re/rnaseq, accessed on 20 February 2025). Quality control assessment of the RNA-seq data was conducted with FASTQC (version 0.11.8) [47] and RSeQC (version 3.0.1) [48]. Reads were mapped to the Homo sapiens reference genome (GRCh37) using the STAR aligner (version 2.6.1d) [49]. Raw gene expression was quantified with featureCounts (version 1.6.4) [50].

Differential expression (DE) was performed with the Nextflow-based rnadeseq pipeline (version 2.0.1, https://github.com/qbic-pipelines/rnadeseq, accessed on 20 February 2025), which integrates the R package DESeq2 (version 1.34.0) [51] for DE analysis in R (version 4.1.2). Genes were considered differentially expressed if the *p*-adjusted value (padj), calculated with the Benjamini–Hochberg method, was <0.05. No logFold change cut-off was applied during the DE analysis assessment. DEG lists were obtained from simple pairwise comparisons using the design formula: ~tissues in DESeq2, in which tissues is the experimental factor/variable with the individual levels of iPSCs, iMSCs, and JPCs. DEG lists were used as input in OmicsPlayground (BigOmics Analytics SA, Lugano, Switzerland) for subsequent pathway enrichment analysis [52].

### 2.10. Statistical Analysis

For the evaluation of MSC and iPSC marker expression, as well as osteogenic markers and Alizarin red quantification, means ± standard deviations were calculated and compared by unpaired Student’s *t*-tests using GraphPad Prism 8.1.0 software. Mean ± standard deviations of senescence marker gene expression and predicted DNAm age were compared by one-way ANOVA (*p* adjusted using Tukey’s multiple comparison test). A *p*-value ≤ 0.05 was considered significant.

## 3. Results

### 3.1. iMSC Characterization

#### 3.1.1. MSC and iPSC Marker Expression

**Figure 1:**
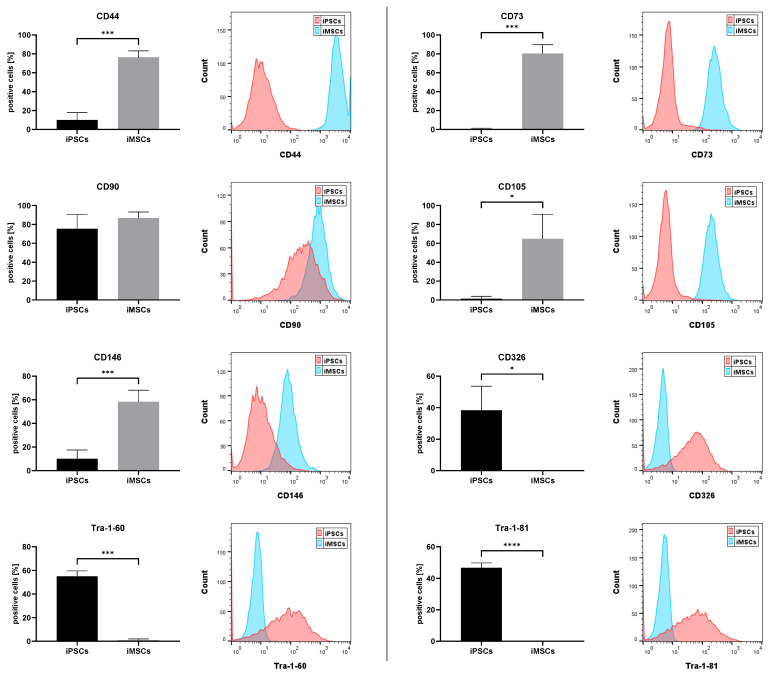
Expression of common MSC and iPSC markers measured by flow cytometry. Expression of MSC (CD44, CD73, CD90, CD105, and CD146) and iPSC surface markers (CD326, Tra-1-60, and Tra-1-81) was measured by flow cytometry in iPSCs (black) and iMSCs (gray) of passage 4. Percentages of positive cells are displayed as means + standard deviation and were compared by unpaired Student’s *t*-tests (n = 4, * *p* < 0.05, *** *p* < 0.001, and **** *p* < 0.0001). Representative flow cytometry histograms with an overlay of iMSCs (blue) and iPSCs (red) are displayed for each surface marker.

Typical MSC and iPSC markers were analyzed by flow cytometry and qPCR to confirm the MSC phenotype of iMSCs after differentiation was completed in passage 4. Figure 1 shows significantly higher percentages of cells expressing MSC surface markers CD44 (*p* = 0.0007), CD73 (*p* = 0.0004), CD105 (*p* = 0.0157), and CD146 (*p* = 0.0007) in iMSCs compared to the originating iPSCs. These levels seem to be slightly lower than usually detected in MSCs, probably due to the intricate fundamental changes during the reprograming and re-differentiation process. CD90 is also strongly expressed in iPSCs and, therefore, shows no significant difference between iPSCs and iMSCs. In addition, a significantly lower percentage of iMSCs express iPSC surface markers CD326 (*p* = 0.0152), Tra-1-60 (*p* = 0.0003), and Tra-1-81 (*p* < 0.0001).

**Figure 2:**
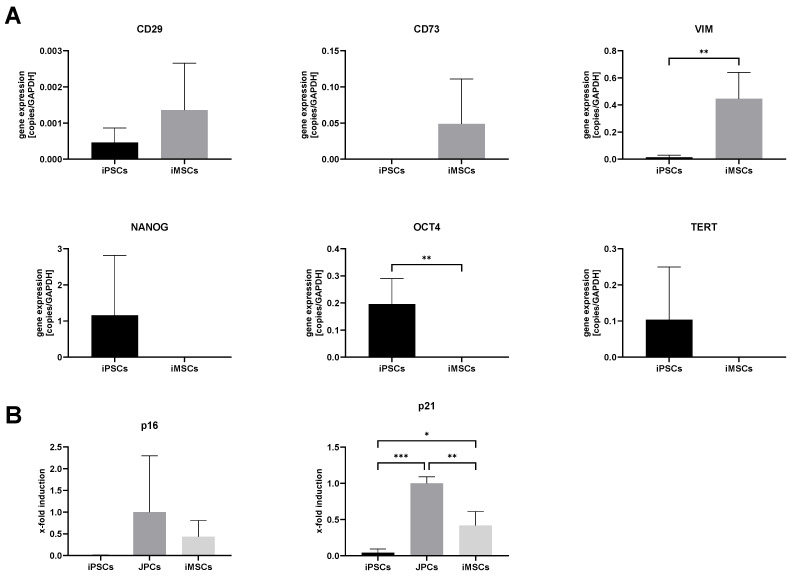
(**A**) Gene expression of common MSC and iPSC markers measured by qPCR. Gene expression of MSC (CD29, CD73, and VIM) and iPSC marker genes (NANOG, OCT4, and TERT) was measured by qPCR in iPSCs (black) and iMSCs (gray) of passage 4. mRNA copy numbers were normalized to the housekeeping gene glycerinaldehyde-3-phosphate dehydrogenase (GAPDH) and means + SD were compared by unpaired Student’s *t*-tests (n = 4, ** *p* < 0.01). (**B**) In addition, expression of senescence marker genes p16 and p21 was quantified by qPCR in iPSCs, iMSCs, and JPCs. mRNA copy numbers were normalized to the housekeeping gene glycerinaldehyde-3-phosphate dehydrogenase (GAPDH) and means + SD (n = 4) were displayed relative to the expression of the JPC samples. Gene expression data were analyzed using one-way ANOVA and Tukey’s multiple comparison test (* *p* < 0.05, ** *p* < 0.01, and *** *p* < 0.001).

The gene expression analysis (Figure 2A) shows a similar pattern, with the upregulation of the MSC markers CD29, CD73, and VIM (*p* = 0.0044), and the downregulation of the iPSC markers NANOG, OCT4 (*p* = 0.0059), and TERT in iMSCs, however, with lesser statistical significance due to higher sample variabilities.

In a previous work of our lab, iMSCs were found to express senescence markers at early passages and have lower growth rates compared to the original JPCs [30]. After the optimization of the differentiation protocol, the gene expression of cellular senescence markers, cyclin-dependent kinase inhibitors p16 and p21, in iMSCs was determined again and compared with the original JPCs and iPSCs (Figure 2B). In comparison to the JPCs and iMSCs, iPSCs showed very low expression levels of the kinase inhibitors. In iMSCs, the p16 and p21 expression is much lower compared to JPCs but higher than in iPSCs. The p21 expression in iMSCs was significantly lower compared to JPCs (*p* = 0.0033) but significantly higher compared to iPSCs (*p* = 0.0262). The p16 expression displayed a similar pattern; however, the differences were not statistically significant due to the higher variation in the JPC group.

#### 3.1.2. Osteogenic Differentiation

**Figure 3:**
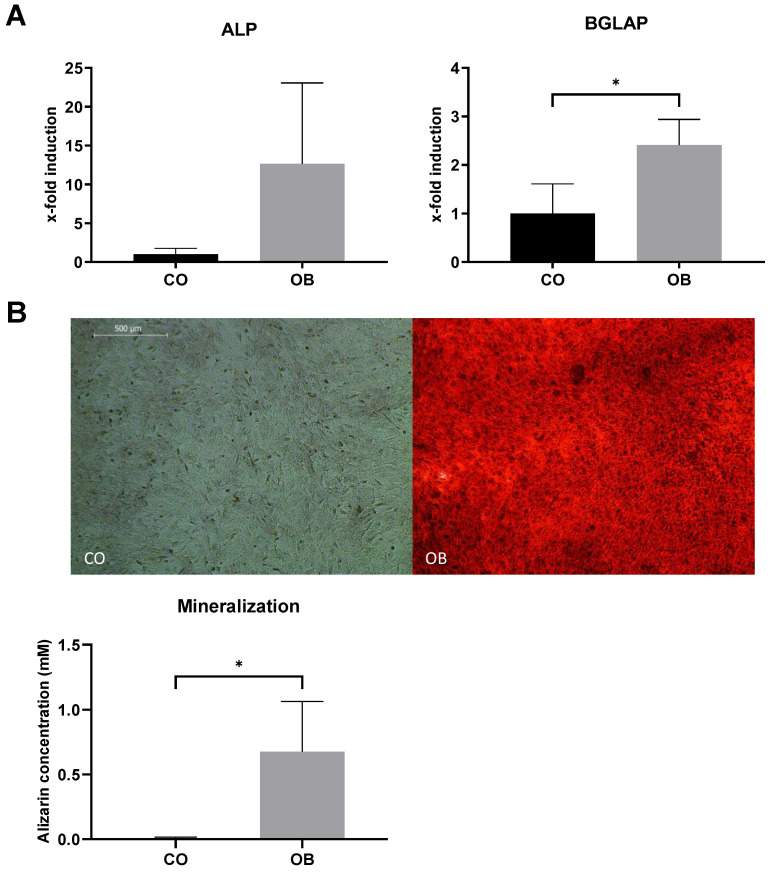
Osteogenic differentiation of iMSCs. (**A**) Expression of osteogenic marker genes (ALP and BGLAP) in iMSCs was quantified by qPCR after 15 days of treatment with control (CO) or osteogenic medium (OB). mRNA copy numbers were normalized to the housekeeping gene glycerinaldehyde-3-phosphate dehydrogenase (GAPDH) and means + SD (n = 4) were displayed relative to the expression of the control sample. (**B**) Mineralization of iMSCs was visualized by Alizarin red staining after 21 days of osteogenic differentiation (representative image, 10x magnification, scale bar = 500 µm). Alizarin concentration was quantified photometrically and means + SD were displayed (n = 4). The mean values of gene expression and Alizarin quantification were compared by unpaired Student’s *t*-tests (* *p* < 0.05).

To confirm the functionality of differentiated iMSCs and their applicability for bone tissue engineering, the cells were osteogenically stimulated in passage 4 after iMSC differentiation was completed. Therefore, iMSCs were incubated in control (CO) or osteoblast medium (OB) for 15–21 days. Osteogenic differentiation was analyzed by qPCR on day 15 by detecting ALP (alkaline phosphatase) and BGLAP (osteocalcin) expression (Figure 3A). Furthermore, Alizarin red staining and quantification of the Alizarin concentration were performed on day 21 as displayed in Figure 3B.

Gene expression analyses showed an upregulation of the osteogenic marker genes ALP (*p* = 0.1176) and BGLAP (*p* = 0.013) in iMSCs during osteogenic differentiation. In addition, Alizarin staining (Figure 3B) showed strong mineralization, and the Alizarin quantification detected significantly higher amounts of calcium phosphate precipitates in osteogenically differentiated iMSCs (*p* = 0.0136).

### 3.2. DNA Methylation

#### 3.2.1. Differential Methylation Analysis

**Figure 4:**
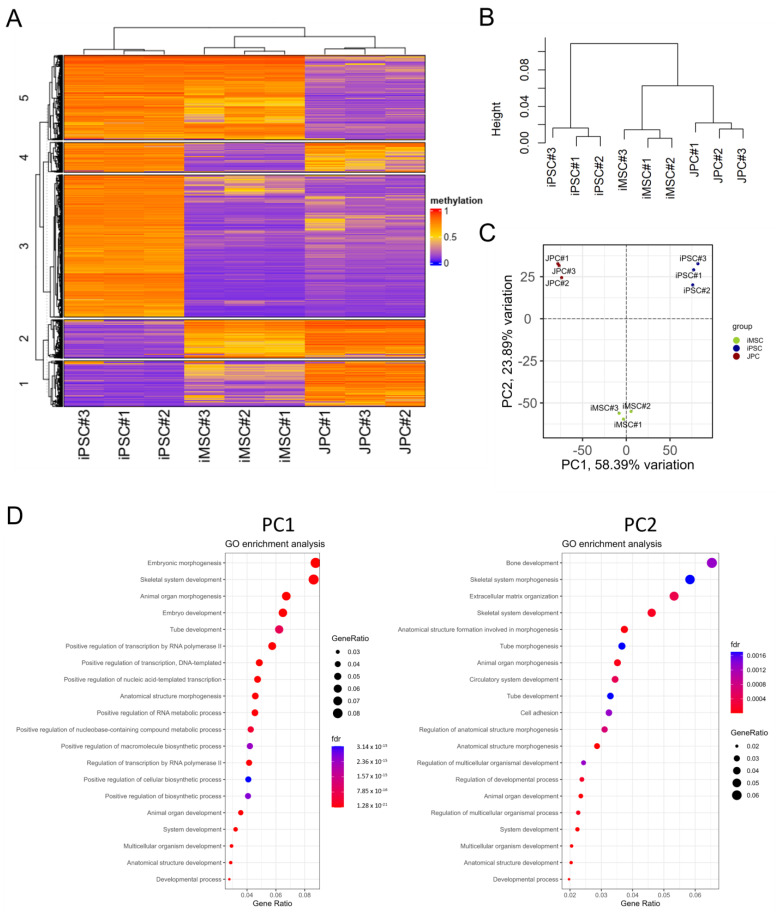
Genome-wide methylation analysis of iMSCs, JPCs, and iPSCs. (**A**) Heatmap of average methylation of the top 20,000 most variable sites. (**B**) Dendrogram from hierarchical clustering of the three cell groups. (**C**) Principal component analysis (PCA) of differential methylation data (blue—JPCs, green—iPSCs, and red—iMSCs). (**D**) Gene ontology (GO) enrichment analysis of 50,000 most variable methylation sites contributing to PC1 and PC2 of the PCA analysis of iPSC, iMSC, and JPC methylation. Dot size represents the ratio of enriched genes associated with each GO biological process. The color of the dots indicates the value of the false discovery rate (fdr).

As epigenetic rearrangements are important events during the differentiation of iPSCs to MSCs, we examined the genome-wide DNA methylation in iMSCs and compared them with JPCs’ and iPSCs’ methylation patterns (Figure 4). Figure 4A depicts a heatmap of the top 20,000 most variable sites, which reveals notable differences between the three cell types. iPSCs exhibit higher numbers of hypermethylated CpGs (left row) compared to JPCs (middle row) and iMSCs (right row). The majority of methylation patterns observed in the top 20,000 most variable sites are shared between iMSCs and JPCs (cluster 2 and 3); yet, the heatmap also displays similarities between iMSCs and iPSCs (cluster 1 and 5), and iMSC-specific methylation patterns (cluster 4). Greater similarities between iMSCs and JPCs are also reflected in the cluster dendrogram (Figure 4B). A distinct trifolious branch representing the three donors can be observed for each of the three cell types. On a higher hierarchical level, iMSCs and JPCs cluster together, displaying a higher degree of similarity compared to iPSCs, which form a separate branch.

The principal component analysis (PCA) shows the clustering of the three cell types in distinct groups (Figure 4C). PC1, representing 58.39% of variation, shows the biggest differences between JPCs and iPSCs, with iMSCs being located in the middle of the PC plot. PC2, which accounts for 23.89% of the total variation, shows clear differences between iMSCs and the other two cell types, suggesting the occurrence of iMSC-specific methylation patterns.

In order to draw conclusions about the functionality of the identified differences in the PCA, the top 50,000 CpGs responsible for the differences in PC1 and PC2 were identified and a gene enrichment analysis was conducted to identify the associated genes. Subsequently, a gene ontology (GO) term enrichment analysis was performed using these gene sets to gain insights into the underlying biological processes (Figure 4D). The dot plot of GO terms of biological processes enriched in PC1 shows many processes involved in embryo development and morphogenesis, which can be attributed to iPSC-specific methylation patterns reflecting the biggest differences to the JPCs they are deriving from. The GO enrichment analysis of PC2 reflects the biggest differences between iMSCs and JPCs/iPSCs. Cellular processes of bone development probably reflecting the differences of iMSCs to iPSCs seem to be involved but with a relatively high false discovery rate (purple fdr). In contrast, processes involved in extracellular matrix organization and cell adhesion show a low fdr and high gene ratios. This result is in line with our experience that iMSCs show a different adhesion behavior compared to JPCs. Further, developmental processes are involved, reflecting the relevant differences of iMSCs compared to JPCs.

To gain more detailed insights, we extracted the genes contributing to the GO terms “bone development”, “skeletal system development”, “skeletal system morphogenesis”, “extracellular matrix organization”, and “cell adhesion”, which are among the most relevant GO terms in PC2, and calculated the average methylation of these genes. Genes showing significant differences between at least two groups are listed in Table 2 and illustrated in diagrams in Supplementary Figure S2. Thereby, methylation patterns specific for iMSCs (COL4A2, FES, LPP, and SORBS3), for JPCs (CD34, CDH13, COL1A1, EPHA2, FMOD, FOXF2, GNAS, ISLR, ITGBR, LRRC17, MGP, PCDHA1, PCDHB15, PCDHGA2, PCDHGA4, SH3PXD2B, SRCIN1, and TGFB3), and for iPSCs (DLC1, EMILIN1, LTBP1, and MYH9) were identified.

From the average methylation states listed in Table 2 and illustrated in Supplementary Figure S2, and based on the general principle that higher methylation (especially in promoter regions) is typically associated with gene silencing, while lower methylation is often associated with higher expression, we can made the following conclusions: (1) iMSCs show more epithelial-like traits; (2) iMSCs deposit less collagen and are less fibrotic; (3) iMSCs show decreased adhesion and a less invasive and motile phenotype; and (4) iMSCs show a lower mesenchymal gene expression compared to the parental JPCs. These findings are in accordance with our previous experience with both cell types.

#### 3.2.2. Enhancer Panel MSCs

The methylation array employed in this investigation comprises multiple CpG sites that do not correlate with the differentiation of MSCs iPSCs. By focusing on MSC-specific (bone marrow (BM)) CpGs, it is possible to make a more precise comparison of significant differences between iMSCs, JPCs, and iPSCs. Consequently, we used a panel of CpGs for BMMSC-specific enhancers, which are regulatory DNA sequences that can enhance the transcription of associated genes, derived from the EnhancerAtlas 2.0 database (Figure 4A,B) [53].

**Figure 5:**
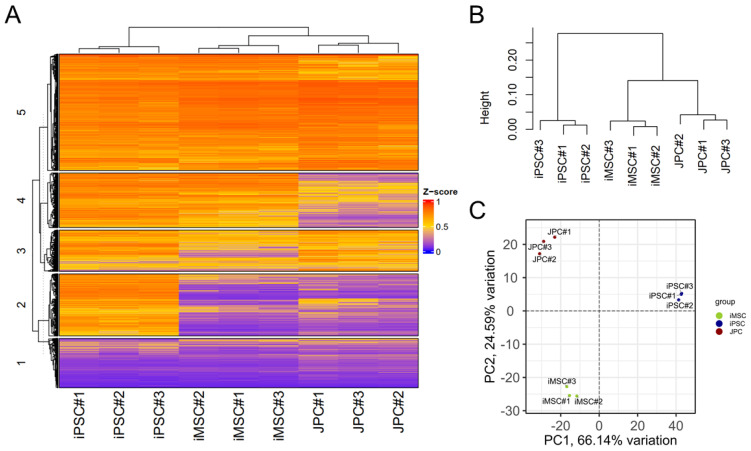
Methylation of BM (bone marrow) MSC-specific enhancers of iMSCs, JPCs, and iPSCs. (**A**) Heatmap of average methylation of the top 20,000 most variable sites in the MSC enhancer set. (**B**) Dendrogram from hierarchical clustering of the three cell groups. (**C**) Principal component analysis (PCA) of differential methylation data (blue—iPSCs, green—iMSCs, and red—JPCs).

The PCA of methylation data of the MSC enhancers (Figure 5C) shows the clustering of the three cell types in distinct groups, as observed in the genome-wide methylation analysis. However, PC1 (66.14% variation) demonstrates a strong similarity between iMSCs and JPCs and more differences between iMSCs and iPSCs. This outcome reflects the functional similarities between iMSCs and JPCs, as shown, for example, in osteogenic differentiation experiments. PC2 (24.59% variation) shows the highest variation between JPCs and iMSCs, with iPSCs being closer to JPCs. This demonstrates again that there is an iMSCs specific methylation pattern to a certain degree.

#### 3.2.3. Age Prediction in iMSCs

**Figure 6:**
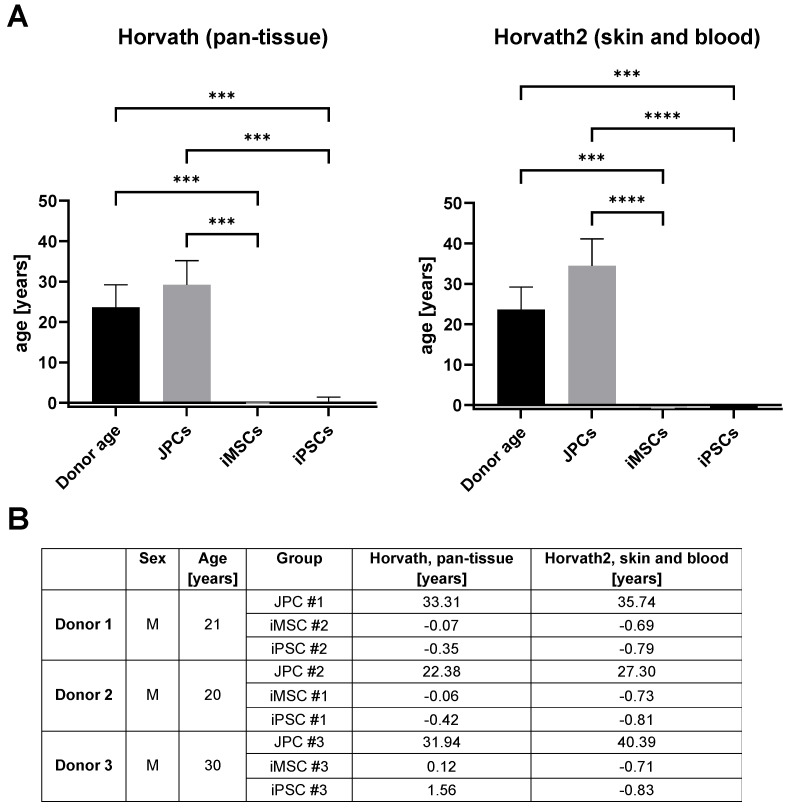
Predicted age of JPCs, iMSCs, and iPSCs compared to the original donor age. (**A**) Methylation based age of the different cell types were calculated with two methylation-based clocks (Horvath pan-tissue and Horvath2, skin and blood) and compared to the original donor age (black) [33]. Mean and SD values are displayed and were compared using one-way ANOVA and Tukey’s multiple-comparisons test (n = 3, *** *p* < 0.001, **** *p* < 0.0001). (**B**) Table of original donor age and predicted DNAm age.

Biological age can be predicted based on DNA methylation (DNAm) clocks, which calculate age based on the methylation of specific CpGs. As these methylation clocks are typically calibrated with DNA from specific sources (most frequently, blood), we employed a range of alternative clocks to enhance the precision of our results (Supplementary Figure S2). The results of the clocks that most accurately reflect the original donor age (23.7 ± 5.5 years) in JPCs (Horvath pan-tissue and Horvath2, skin and blood) are displayed in Figure 6. The Horvath pan-tissue clock calculated an average age of 29.2 ± 6.0 years for the JPCs, 0.0 ± 0.1 years for iMSCs, and 0.3 ± 1.1 years for iPSCs. The Horvath2, skin and blood clock calculated an average age of 34.5 ± 6.6 years for the JPCs, −0.7 ± 0.0 years for iMSCs, and −0.8 ± 0.0 years for iPSCs. Both clocks show a significant reduction in methylation age following the reprogramming of JPCs into iPSCs. This finding is consistent with previous data from Horvath (2013) which demonstrated that embryonic stem cells have a DNAm age close to zero, indicating the epigenetic rejuvenation of reprogrammed JPCs [33]. Furthermore, the subsequent differentiation of iPSCs into iMSCs did not result in a notable increase in the DNAm age of iMSCs compared to iPSCs, indicating that the rejuvenation effect was maintained.

### 3.3. Transcriptome Analysis

**Figure 7:**
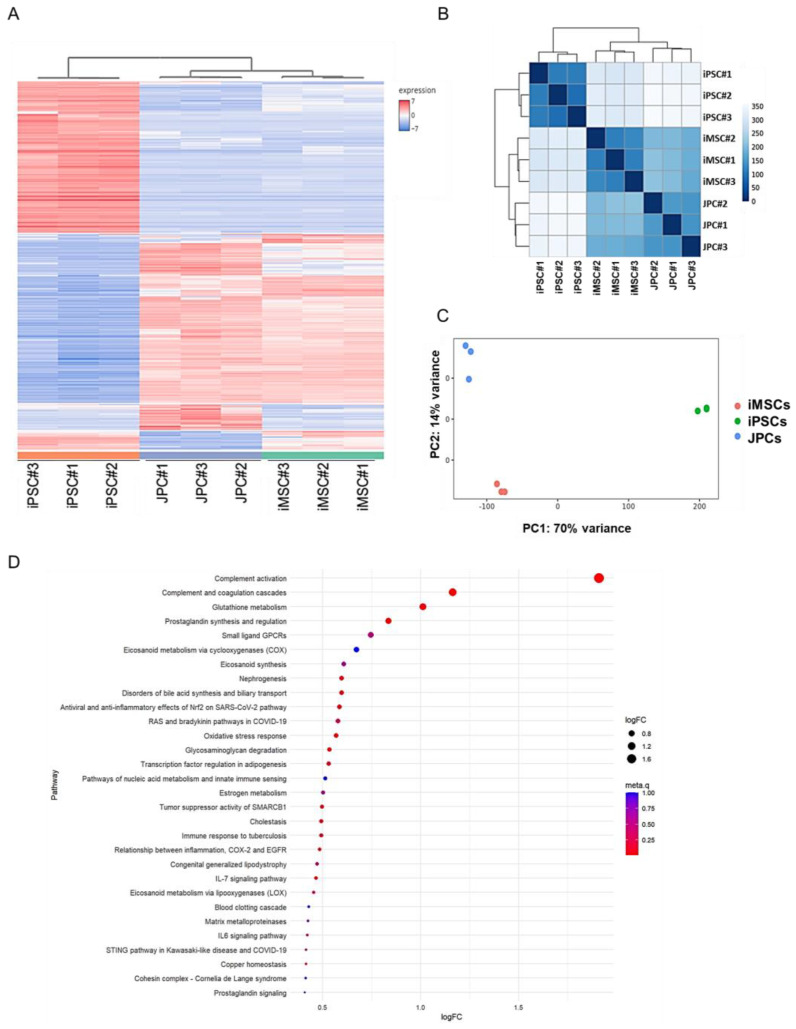
Transcriptome analysis of iMSCs, JPCs, and iPSCs. (**A**) Heatmap of the top 500 differentially expressed genes in iPSCs, JPCs, and iMSCs. (**B**) Heatmap and dendrogram from hierarchical clustering of the three cell groups. (**C**) Principal component analysis (PCA) of differential gene expression data (red—iMSCs, green—iPSCs, and blue—JPCs). (**D**) Pathway analysis of differentially expressed genes in JPCs vs. iMSCs. Dot size represents the logarithmic fold change (logFC) of genes associated with each WikiPathways GO term. The color of the dots indicates the meta.q value.

In comparison with DNA methylation, transcriptome analysis has the capability to provide a more comprehensive insight into the functional differences between different cell types (Figure 7). Figure 7A presents a heatmap of the top 500 differentially expressed genes, which are divided into four clusters. The two largest clusters, which account for over 80% of the data, differ between iPSCs and the two other groups. A smaller proportion of differentially expressed genes is shared between iPSCs and iMSCs, while, in contrast to data obtained from methylomics, no cluster of iMSC-specific genes was observed.

The distance heatmap (Figure 7B) further illustrates the greater similarity between iMSCs and JPCs. It is evident that the distances between iMSCs and JPCs are less pronounced compared to those between iPSCs and the other groups. This observation is further substantiated by the dendrogram, which clustered iMSCs and JPCs at a higher hierarchical level compared to iPSCs, which formed a separate branch.

PCA further substantiates this observation, demonstrating a distinct clustering of the three cell types (Figure 7C). PC1, which accounts for 70% of the variation, shows JPCs and iMSCs opposite to iPSCs, with iMSCs slightly closer to the right than JPCs. PC2, which accounts for 14% of the total variation, demonstrates differences between iMSCs and the other two cell types, with iPSCs closer to iMSCs than JPCs.

To draw conclusions about functional differences between iMSCs and JPCs, a pathway analysis of differentially expressed genes in iMSCs and JPCs was conducted and displayed in Figure 7D. Thereby, only the first four listed pathways, complement activation (metaq = 0.009013), complement and coagulation cascades (metaq = 0.014027), glutathione metabolism (metaq = 0.044512), and prostaglandin synthesis and regulation (metaq = 0.015677), reached significant values and were shown to be elevated in JPCs compared to iMSCs.

### 3.4. Teratoma Formation

**Figure 8:**
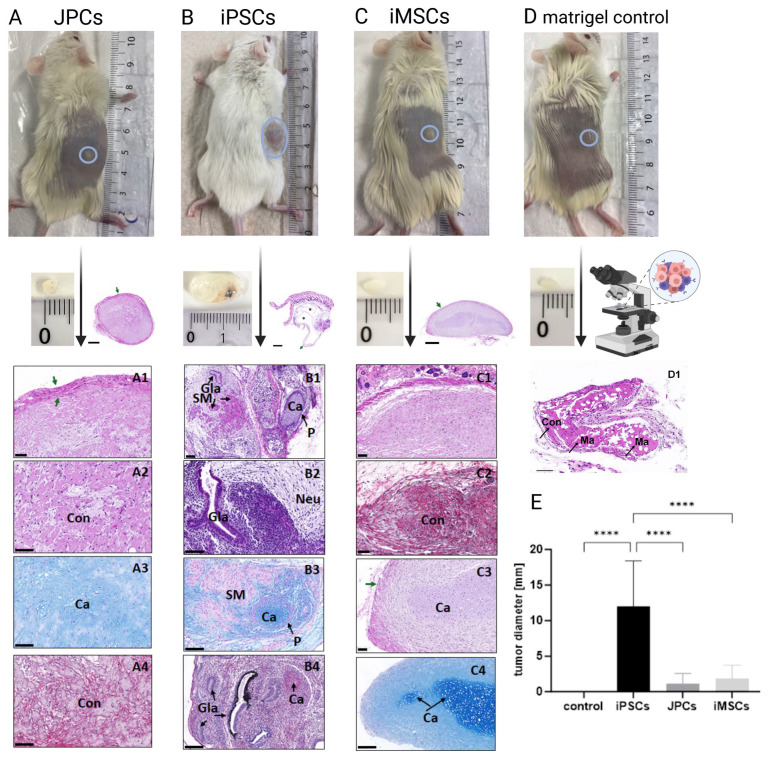
Histological and histochemical analyses of teratoma and mass formation by subcutaneously injected JPCs, iPSCs, and iMSCs (after 8 weeks). Representative low-magnification HE images are included for each condition, displaying the formed mass and its surrounding capsules (green arrows). (**A**) The JPC group was characterized by the formation of a solid connective tissue (Con) mass surrounded by a collagenous capsule (green arrows). In higher magnification images, fibroblast-like cells immersed in a highly dense extracellular matrix can be observed (HE = A1 and A2), showing a positive AB histochemical reaction for proteoglycans (A3 = turquoise reaction) and fibrillar collagens (A4 = FMPS red histochemical reaction for collagen). (**B**) The iPSC group exhibited well-differentiated teratomas with cystic areas (see * in HE low-magnification image). In addition, cartilaginous (Ca) and perichondrium (P), smooth muscle (SM), neural (Neu), and various epithelial structures (glands (Gla)) were clearly identified confirming that these teratomas contain elements from the three germ layers (HE = B1 and B2; AB = B3). Moreover, pigmented epithelial structures were also observed and confirmed by FMPS histochemical method which stained collagen red and melanin as a black precipitate (B4). (**C**) The iMSC group showed solid mass with similar features to the JPC group (HE = C1 and FMPS = C2), but also displayed chondrogenic structures without perichondrium (HE = C3) with a highly positive reaction for AB (C4). Scale bar: 500 µm for low-magnification images; 100 µm for higher-magnification images. (**D**) Matrigel control (scale bar 100 µm) showing (**D1**) connective tissue (Con) and Matrigel compounds (Ma). (**E**) The resulting tumor size developed after 8 weeks in animals receiving Matrigel only or Matrigel plus iPSCs, JPCs, and iMSCs (50 µL/50 µL) is illustrated. **** *p* < 0.0001.

Eight weeks after the subcutaneous injection of JPCs, iPSCs, and iMSCs, teratoma and mass formation were examined macroscopically and histologically (Figure 8). The size of the entities formed varied among groups; those derived from the iPSC group were the largest, reaching up to 18 mm and showing significant differences to the compared three groups (*p* < 0.0001, Figure 8B,E). In addition, histological analyses confirmed the mass formation in all groups with clear histological differences (Figure 8, lower panel). The histological staining of tissues derived from JPC injection confirmed the neoformation of a solid-connective-tissue-derived tumor or mass. The mass was well-delimited by a continuous connective capsule and was composed of an irregular and dense extracellular matrix and fibroblasts (mesodermal origin). Histochemistry showed a clear positive reaction for proteoglycans and fibrillar collagen fibers (Figure 8(A3,A4)).

When iPSC-derived tumors were analyzed, the histology clearly confirmed teratoma formation. These teratomas showed cystic and solid areas where it was possible to identify well-differentiated histological structures that derived from the three germ layers (ectoderm, mesoderm, and endoderm). Indeed, nervous-tissue-like areas (Figure 8(B2)), diverse epithelial components (simple, pseudostratified ciliated epithelium, prismatic epithelium with goblet cells, and even pigmented epithelial structures; Figure 8(B1,B2,B4)), hyaline cartilage (with perichondrium; Figure 8(B3,B4)), and smooth muscle arrangements (Figure 8(B1,B3)) were clearly identified by HE and histochemical methods.

Interestingly, when iMSC-derived tumors were evaluated, histological staining confirmed the formation of a connective tissue mass (mesodermal origin) comparable to those formed by JPCs. However, in one animal, the formed mass contained areas with hyalin chondrogenic differentiation, but without perichondrium (Figure 8(C3,C4)).

Finally, the analysis of the injected Matrigel confirmed the presence of the implanted hydrogel which was surrounded by normal connective tissue elements and partially recellularized. No inflammatory host reaction was observed around the tumors tested as expected. While 12 of the 15 iPSC-injected mice, 6 of the 15 JPC-injected animals, and 8 of the 15 iMSC-injected mice developed entities at the injection site, those from the iPSC-injected mice were significantly larger (Figure 8A–C,E).

## 4. Discussion

iMSCs represent a valuable cellular source for future regenerative therapies. In contrast to adult MSCs, they can be produced in sufficient quantity and with consistent quality [54]. To date, 13 clinical trials have utilized pluripotent-stem-cell-derived MSCs (6 using iPSCs and 7 using ESCs) for the treatment of a wide variety of diseases [9]. In seven of these trials, the immunomodulatory effects of MSCs are exploited, for instance, to treat GvHD, multiple sclerosis, and other diseases. Six trials are in the field of regenerative medicine, testing treatments for non-healing diabetic foot ulcers, meniscus injury, osteoarthritis, pulmonary fibrosis from COVD-19, primary ovarian insufficiency, and intrauterine adhesions. These studies demonstrate the broad application spectrum of iMSCs, similar to that of adult MSCs, underscoring their significant therapeutic potential.

However, until iMSC products are ready for the market, several challenges have to be resolved. Of primary concern is a potential tumorigeneity due to the teratoma formation potential of undifferentiated iPSCs. However, the safety of iMSCs has already been demonstrated in numerous studies, including the one presented here. In addition, the choice of cell source is discussed due to the potential epigenetic memory that somatic cells retain in iPSCs [12]. Furthermore, the functional differences between iMSCs generated using varying differentiation protocols are poorly understood due to a lack of comparative studies [13].

Our iMSC differentiation protocol is based on the spontaneous differentiation in MSC medium and the subsequent selection of MSC-like cells (CD105 positivity), which is in contrast to some protocols that attempt to mimic the stepwise differentiation of MSCs during embryonic development by the sequential addition of various growth factors or inhibitors [55]. As a consequence, there is a possibility of impurity within the cell population and the presence of other MSC-like cells, such as fibroblasts. However, as mentioned in the introduction part, the generated iMSCs possess the required tri-lineage stem cell potential. Further, teratoma formation testing in NOG mice demonstrated the absence of undifferentiated iPSCs, as no teratoma formation was found in iMSC-injected mice, indicating the safety of the produced cells. Even in clinical studies, where iMSCs have been injected into humans to treat acute graft-versus-host disease (GvHD), their safety has been proven [56].

The phenotype of a particular cell type is determined by its distinct transcriptional profile, which is dictated by epigenetic modifications such as DNA methylation [57]. Epigenetic rearrangements are particularly evident during stem cell differentiation, when the transcriptional profiles of cells undergo fundamental changes. Having taken this into account, we performed a DNA methylation analysis to gain further insight into the similarities and differences between the three cell types, iMSCs, iPSCs, and the original JPCs.

Our data showed that all cell types exhibited distinct methylation profiles, as visualized by the principal component analysis (PCA). A gene ontology (GO) enrichment analysis was conducted on the most variable methylation sites contributing to principal components PC1 and PC2 to gain further insight into the biological processes affected by these methylation patterns. In PC1, where the biggest differences were observed between JPCs and iPSCs, processes related to embryo development and morphogenesis were among the most relevant GO terms. This can be attributed to the distinct methylation characteristics of iPSCs. Interestingly, in PC2, the most relevant GO terms were related to bone development, skeletal system development, and extracellular matrix organization. Here, the biggest differences were found between iMSC on one site and JPC and iPSCs on the other site. However, the hierarchical clustering of methylation profiles demonstrated the clustering of iMSCs and JPCs on a higher level, displaying a greater similarity compared to iPSCs.

Further, we focused the analysis on MSC-specific enhancers [53], as the majority of the examined CpGs are not directly involved in the differentiation of the investigated MSCs. Including this approach, we found that JPCs and iMSCs exhibit strong similarities, as demonstrated by the performed PCA analysis. This outcome reflects the functional similarities between iMSCs and JPCs, as shown, for example, in osteogenic differentiation experiments. However, differences were identified also between iMSCs and JPCs, demonstrating iMSC-specific methylation patterns. The interpretation of the data regarding the cause of these differences is challenging, as there are several possible explanations. These include the presence of incompletely differentiated cells, cells that have undergone differentiation into a different cell type, or patterns related to cellular reprogramming. But primary MSCs derived from different tissues were also shown to exhibit notable differences in their methylation profiles, which could contribute to the detected differences [58,59,60]. At the transcriptome level, Wruck et al. also identified significant differences between MSCs derived from various sources and postulated that these differences are mainly driven by the microenvironment and epigenetic patterns that are altered by aging [54]. Moreover, the clustering of iMSCs and young MSCs (obtained from the umbilical cord) was demonstrated, and a rejuvenated transcription profile of iMSCs was observed [54]. Similarly, to gain further insight into the significance of the identified differences in DNA methylation, it is necessary to compare our data with datasets of MSCs from diverse origins and donor ages, as well as other cell types, such as fibroblasts and endothelial cells. Since we used the CpG panel of BMMSCs from the enhancerAtlas 2.0 for comparison, the consideration of BMMSC samples would have been advantageous, indicating a limitation of our study.

We also employed the DNA methylation data to analyze the biological age of the generated iMSCs, thereby demonstrating epigenetic rejuvenation. We used various epigenetic clocks (Supplementary Materials), including the Horvath pan-tissue and Horvath2 skin and blood clocks, which most accurately reflected the chronological age of the donors of the original JPCs [33], resulting in an estimated age of approximately zero years of iMSCs and iPSCs. This result demonstrates that the reprogramming of JPCs results in epigenetic rejuvenation, a phenomenon observed previously also by other researchers [33]. Furthermore, this rejuvenation is maintained during iMSC differentiation, raising the possibility that iMSCs could harbor improved regenerative capacities. In the current work, we compared the osteogenic potential of JPCs and iMSCs by focusing on their biomechanical signatures [61]. We detected differences in cellular stiffnesses of JPCs compared to iMSCs but the same reduction in cellular stiffness during the process of osteogenesis and a very similar hardness of formed calcium phosphate precipitates. On the other site, compared to the JPC predecessors, we identified a resistance to hypoxic conditions in iMSCs, a characteristic which can be quite relevant for their future clinical applications.

Epigenetic changes represent only one of the 12 hallmarks of aging postulated by López-Otín et al. and, thus far, no direct consequence of age-related DNA methylation on cellular function has been demonstrated [28]. On the other hand, in experiments with partial reprogramming, a transient expression of reprogramming factors after which cells subsequently revert to their original differentiated state, improvements in the repair mechanisms of tissues and cells have been observed, both in vitro and in vivo [62,63,64,65,66]. These improvements were accompanied by the restoration of youthful DNA methylation patterns. Furthermore, the transient reprogramming of genetically modified animals has been shown to extend the lifespan of progeroid mice. It thus appears reasonable to posit that reprogramming enables the generation of iMSCs with an enhanced regenerative capacity relative to primary MSCs derived from the same donor.

**Figure 9:**
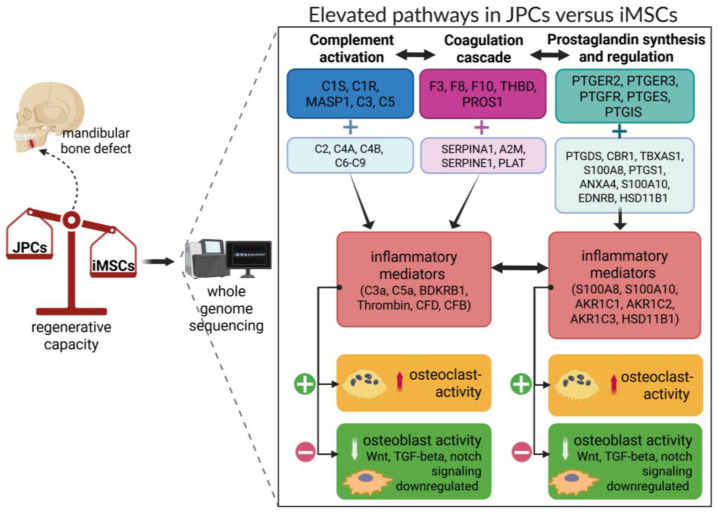
Hypothesis of possible implications based on elevated pathways JPCs on a potentially higher bone regenerative capacity of iMSCs compared to JPCs. As illustrated in Figure 7D, the highest differences detected by transcriptomics (WikiPathways analysis) in JPCs versus iMSCs were referred to the activation of the complement and coagulation cascades, glutathione metabolism, and prostaglandin synthesis and regulation. Although the mentioned pathways play crucial roles in bone development, their chronic activation leads to higher osteoclasts activity and inhibition of osteoblasts. Considering the inflammatory mediators of these pathways, we assume that iMSCs create an environment which balances a higher regenerative capacity than their predecessors.

When transcriptomics is taken into account and we zoom into the four significantly elevated pathways in JPCs compared to iMSCs, we can speculate about their biological relevance, especially with the focus on the resulting bone regenerative capacity (Figure 9). Complement factor C5 and its cleavage products are essential for bone healing. Mice lacking C5 exhibited impaired healing, suggesting that C5 and its cleavage products, C5a and C5b, are necessary for normal healing processes [67]. Research has indicated a significant upregulation of the anaphylatoxin receptors C5aR and C3aR during the differentiation of mesenchymal stem cells into osteoblasts, with C3a and C5a promoting this process [67]. Osteoclast maturation depends on C3, C3aR, and C5aR. C5a, a potent pro-inflammatory molecule, amplifies inflammatory signals, including IL-6 and TNF-α [68]. A transcriptomic analysis in JPCs revealed an elevated expression of genes related to complement and coagulation cascades, as well as IL-6 and stimulator of interferon genes (STING) signaling pathways, which are associated with inflammatory bone diseases such as rheumatoid arthritis and osteoarthritis [67].

C3a und C5a can activate thrombocytes, and thrombin, in turn, activates C3 and C5, with both cascades mutually reinforcing themselves. The coagulation cascade influences bone remodeling via thrombin, which activates RANKL and inhibits OPG. The plasminogen activator inhibitor-1 SERPIN1 (SerpinE1, PAI-1) negatively affects bone metabolism [69], whereas SERPINA1 (alpha1-antitrypsin, AAT) has anti-inflammatory properties [70]. Increased prostaglandin synthesis and regulation pathways were found in JPCs compared to iMSCs. A continuous treatment with PGE2 was shown to impair matrix mineralization by hBMMSCs and to promote an adipogenic phenotype at the same time. The inhibiting effects of PGE2 of hBMMSC-mediated matrix mineralization are thought to be mediated primarily through PGE2 receptors EP2 and EP4 [71]. Indeed, we made the observation that untreated JPCs are more prone to build lipid droplets during in vitro culturing as untreated iMSCs and PTGER2, but not PTGER4, was shown to be elevated in JPCs.

Annexins and S100 proteins play a role in mineralization. Research showed that S100A10 was selectively translocated to matrix vesicles of Saos-2 cells upon mineralization [72], whereas, in the context of cancer, S100A10 was shown to be linked to tumor progression [73]. Data obtained from studies with RA patients revealed that systemic and synovial expression levels of S100A8/A9 correlated with joint inflammation and damage in arthritis [74]. AKR1C1 belongs to a superfamily of NADPH-dependent reductases that convert steroid hormones and endogenous prostaglandins. Research demonstrated that AKR1C1 acts as a negative regulator of osteogenesis and AKR1C2 promotes adipogenesis [75,76]. Glutathione protects against oxidative stress and is required for the synthesis of leukotrienes and prostaglandins, whereby the reduced form GSH neutralizes ROS while the oxidized form GSSH has an activating effect on osteoclasts [77].

In summary, we assume that the identified significantly activated complement, coagulation cascades, and elevated prostaglandin synthesis, as well as IL-6/STING signaling pathways (not significant) combined with downregulated Wnt, TGF-β, and Notch signaling (not significant) in JPCs, may rather favor bone resorption compared to iMSCs. Therefore, we hypothesize that the herein generated and rejuvenated iMSCs may have a higher regenerative capacity than the parental JPCs. However, future in vivo studies have to validate this hypothesis.

## 5. Conclusions

In the present study, we demonstrated a methylation profile of iMSCs close by that of JPCs on the one site, and a distinct iMSC-specific methylation pattern on the other site. The application of DNA methylation (DNAm) clocks showed a dramatic reduction in DNAm age to approximately zero in iMSCs, similar to iPSCs. This profound reset in biological age and the performed transcriptome analyses suggest that the herein generated iMSCs could possess an enhanced regenerative capacity compared to the parental JPCs. Future in vivo bone defect model studies should test their efficacy and the validity of our hypothesis.

Despite the extensive number of clinical studies (1476 by 2023) conducted with adult MSCs, only one FDA-approved product has emerged from these studies [78,79]. An increased efficacy benefiting from iMSCs rejuvenation could provide the necessary incentive for stem cell therapies to finally achieve a breakthrough in clinical applications.

## Figures and Tables

**Table 1:** List of antibodies (antigen, isotype, conjugate, and manufacturer) used for flow cytometry.

Human Antigen	Isotype	Conjugate	Company
SSEA4	human recombinant antibody (REA)	PE	Miltenyi, Bergisch Gladbach, Germany
TRA-1-60		PE	
TRA-1-81		PE	
REA-Isotype		PE	
CD73	mouse IgG1	PE	BD Biosciences, Franklin Lakes, NJ, USA
CD90		PE	
CD105		APC	BioLegend, San Diego, CA, USA
IgG1-Isotype		APC	
IgG1-Isotype		PE	R&D Systems, Minneapolis, MN, USA

**Table 2:** Enriched genes contributing to GO terms “bone development”, “cell adhesion”, “extracellular matrix organization”, “skeletal system development”, and “skeletal system morphogenesis”. The table displays average methylation of JPCs, iMSCs, and iPSCs, as well as *p* values of pairwise comparisons of the three groups carried out by repeated-measures one-way ANOVA and Tukey’s multiple comparison test. ECM = extracellular matrix, SSD = skeletal system development, SSM = skeletal system morphogenesis.

Genes	Average Methylation			*p*-Value			GO Terms				
	JPCs	iMSCs	iPSCs	JPCs vs. iMSCs	JPCs vs. iPSCs	iMSCs vs. iPSCs	Cell Adhesion	Bone Development	ECM	SSD	SSM
CCDC80	0.32 ± 0.2	0.49 ± 0.33	0.69 ± 0.15	0.0003	<0.0001	0.001			X		
CD34	0.43 ± 0.28	0.54 ± 0.29	0.55 ± 0.27	<0.0001	0.0034	0.9671	X				
CDH13	0.56 ± 0.2	0.63 ± 0.27	0.67 ± 0.2	<0.0001	<0.0001	0.0079	X				
CFDP1	0.45 ± 0.27	0.49 ± 0.31	0.56 ± 0.28	0.1963	0.0022	0.1178	X				
COL1A1	0.23 ± 0.12	0.57 ± 0.33	0.59 ± 0.29	<0.0001	<0.0001	0.5364		X	X	X	X
COL4A2	0.76 ± 0.19	0.69 ± 0.25	0.74 ± 0.16	<0.0001	0.3314	0.0006			X		
DDR2	0.5 ± 0.31	0.61 ± 0.29	0.73 ± 0.09	0.0027	<0.0001	0.0091	X		X		
DLC1	0.53 ± 0.3	0.5 ± 0.3	0.63 ± 0.26	0.2942	<0.0001	<0.0001	X				
EFNA5	0.61 ± 0.28	0.53 ± 0.29	0.57 ± 0.27	<0.0001	0.2943	0.1527	X				
EMILIN1	0.42 ± 0.32	0.44 ± 0.34	0.61 ± 0.21	0.4037	0.0121	0.0332	X		X		
EPHA2	0.59 ± 0.32	0.48 ± 0.35	0.53 ± 0.32	0.0002	0.0226	0.0669				X	
FES	0.53 ± 0.28	0.68 ± 0.21	0.6 ± 0.25	<0.0001	0.0276	<0.0001	X				
FMOD	0.52 ± 0.3	0.7 ± 0.13	0.64 ± 0.19	0.0044	0.0374	0.1394			X		
FOXF2	0.57 ± 0.3	0.19 ± 0.1	0.12 ± 0.05	<0.0001	<0.0001	0.0011			X		
GNAS	0.59 ± 0.22	0.4 ± 0.26	0.38 ± 0.25	<0.0001	<0.0001	<0.0001	X	X		X	X
ISLR	0.45 ± 0.24	0.76 ± 0.13	0.72 ± 0.11	<0.0001	<0.0001	0.1003	X				
ITGBL1	0.58 ± 0.27	0.71 ± 0.14	0.72 ± 0.13	<0.0001	0.0005	0.8764	X				
LPP	0.67 ± 0.24	0.65 ± 0.24	0.69 ± 0.18	0.0731	0.2886	0.0056	X				
LRRC17	0.42 ± 0.25	0.51 ± 0.31	0.63 ± 0.18	0.0803	0.0021	0.168		X		X	
MGP	0.4 ± 0.28	0.73 ± 0.09	0.72 ± 0.12	0.0047	0.0056	0.9947				X	X
MYH9	0.61 ± 0.26	0.62 ± 0.28	0.69 ± 0.21	0.6502	0.0001	0.0016	X				
PCDHA1	0.47 ± 0.17	0.68 ± 0.16	0.66 ± 0.18	<0.0001	<0.0001	0.6186	X				
PCDHB15	0.33 ± 0.14	0.62 ± 0.16	0.56 ± 0.2	<0.0001	<0.0001	0.0675	X				
PCDHGA2	0.46 ± 0.21	0.68 ± 0.2	0.68 ± 0.2	<0.0001	<0.0001	0.9797	X				
PCDHGA4	0.44 ± 0.2	0.71 ± 0.21	0.65 ± 0.24	<0.0001	<0.0001	<0.0001	X				
SH3PXD2B	0.57 ± 0.29	0.68 ± 0.28	0.68 ± 0.24	<0.0001	<0.0001	0.9922		X	X	X	
SORBS3	0.46 ± 0.31	0.52 ± 0.31	0.45 ± 0.29	0.005	0.7766	0.0007	X				
SRCIN1	0.57 ± 0.23	0.41 ± 0.29	0.39 ± 0.3	<0.0001	<0.0001	0.2304	X				
TGFB3	0.37 ± 0.29	0.54 ± 0.3	0.52 ± 0.29	0.0056	0.0102	0.6925		X		X	X

## Data Availability

The datasets used and/or analyzed during the current study are available from the corresponding author upon reasonable request.

## Supplementary Materials

The following supporting information can be downloaded at: https://www.mdpi.com/article/10.3390/cells14090627/s1, Figure S1: Experimental procedure to test the ability to induce teratoma formation. Figure S2: Enriched genes contributing to GO terms “bone development”, “cell adhesion”, “extracellular matrix organization”, “skeletal system development”, and “skeletal system morphogenesis”. Table S1: Age prediction (years) using listed methylation-based clocks (trained for different tissues) compared to the chronological age. Table S2: List of involved WikiPathways extracted from the top 500 differentially expressed genes in JPCs versus iMSCs.

## Abbreviations

The following abbreviations are used in this manuscript:

BcM	B18R conditioned medium
DNAm	DNA methylation
ECM	extracellular matrix
fdr	false discovery rate
GO	gene ontology
GvHD	graft-versus-host disease
HE	hematoxylin–eosin
hPL	human platelet lysate
iPSCs	induced pluripotent stem cells
iMSCs	iPSC-derived mesenchymal stem cells
JPCs	jaw periosteal cells
MSCs	mesenchymal stem cells
PCA	principal component analysis
SASP	senescence-associated secretory phenotype
NaB	sodium butyrate
VTN	vitronectin
