# Supplementary material for: Revitalizing the Epigenome of Adult Jaw Periosteal Cells: Enhancing Diversity in iPSC-Derived Mesenchymal Stem Cells (iMSCs)
Source: Cells. 2025 Apr 22;14(9):627. doi: 10.3390/cells14090627 (PMC12071994; doi:10.3390/cells14090627)
Supplement: Supplementary file 1 [file cells-14-00627-s001.zip › cells-3517602-supplementary.pdf]

cells

MDPI

Revitalizing the Epigenome of Adult Jaw Periosteal Cells: Enhancing Diversity in iPSC-derived mesenchymal stem cells (iMSCs)

Felix Umrath [1,2], Valerie Wendt [1], Gilles Gasparoni [3], Yasser Narknava [1], Jörn Walter [3], Bernd Lethaus [1], Josefin Weber [4], Victor Carriel [5], Meltem Avci-Adali [4] and Dorothea Alexander [1,*]

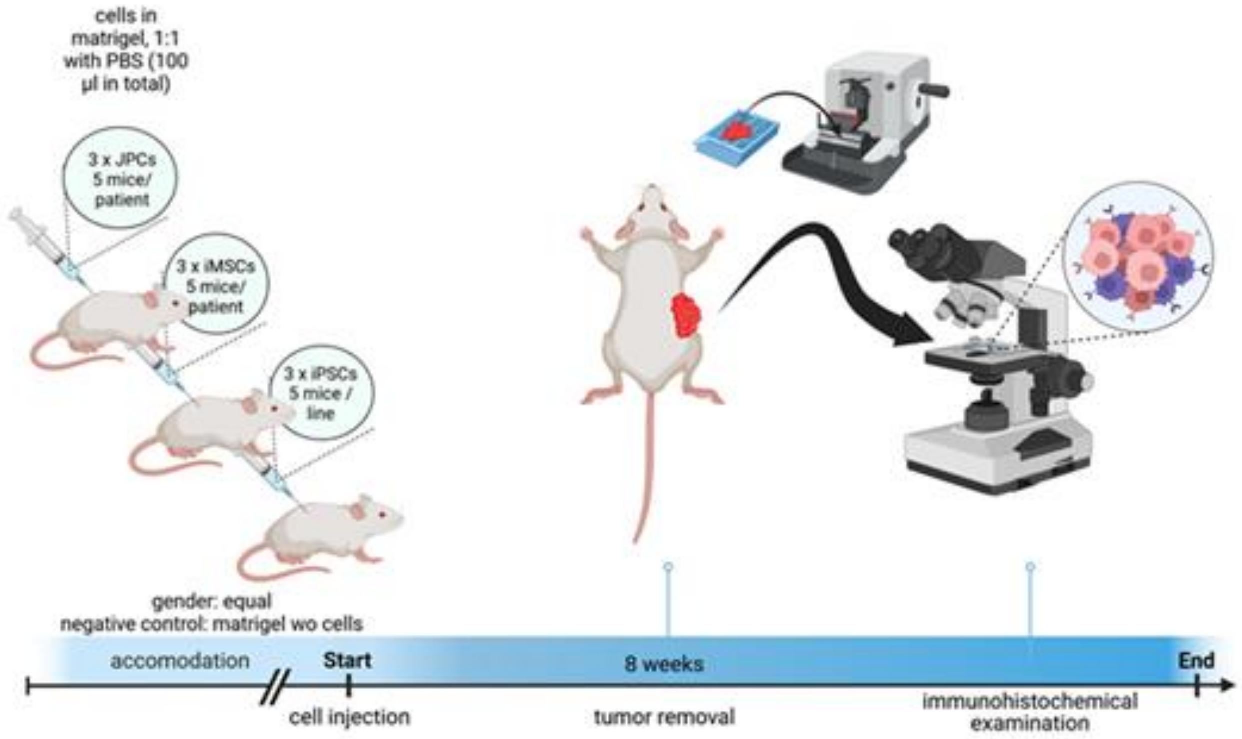

Supplementary Figure S1. Experimental procedure to test the ability to induce teratoma formation. All three cell types (JPCs, iMSCs, iPSCs, derived from 3 human donors) were mixed (1:1) into a matrigel solution and injected subcutaneously into immunodeficient mice (n = 5 per group). After 8 weeks, the mice were sacrificed and isolated samples were examined histologically.

 https://doi.org/10.3390/xxxxx

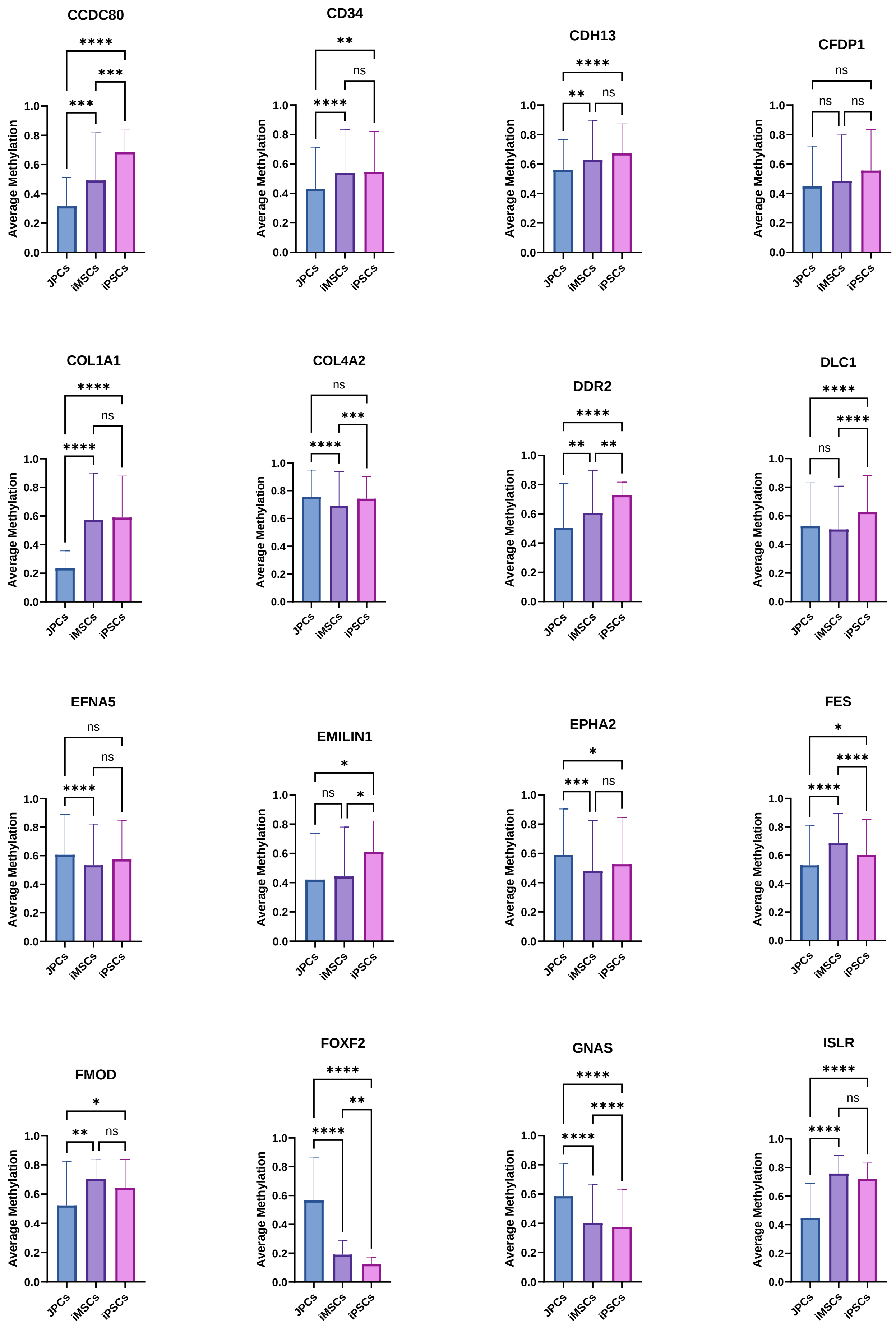
CCDC80
****
***
***
CD34
**
ns
****
CDH13
****
**
ns
CFDP1
ns
ns
ns
COL1A1
****
ns
****
COL4A2
ns
***
****
DDR2
****
**
**
DLC1
****
****
ns
EFNA5
ns
ns
****
EMILIN1
*
ns
*
EPHA2
*
***
ns
FES
*
****
****
FMOD
*
**
ns
FOXF2
****
**
****
GNAS
****
****
****
ISLR
****
ns
****
Average Methylation
0.0
0.2
0.4
0.6
0.8
1.0
JPCs
iMSCs
iPSCs

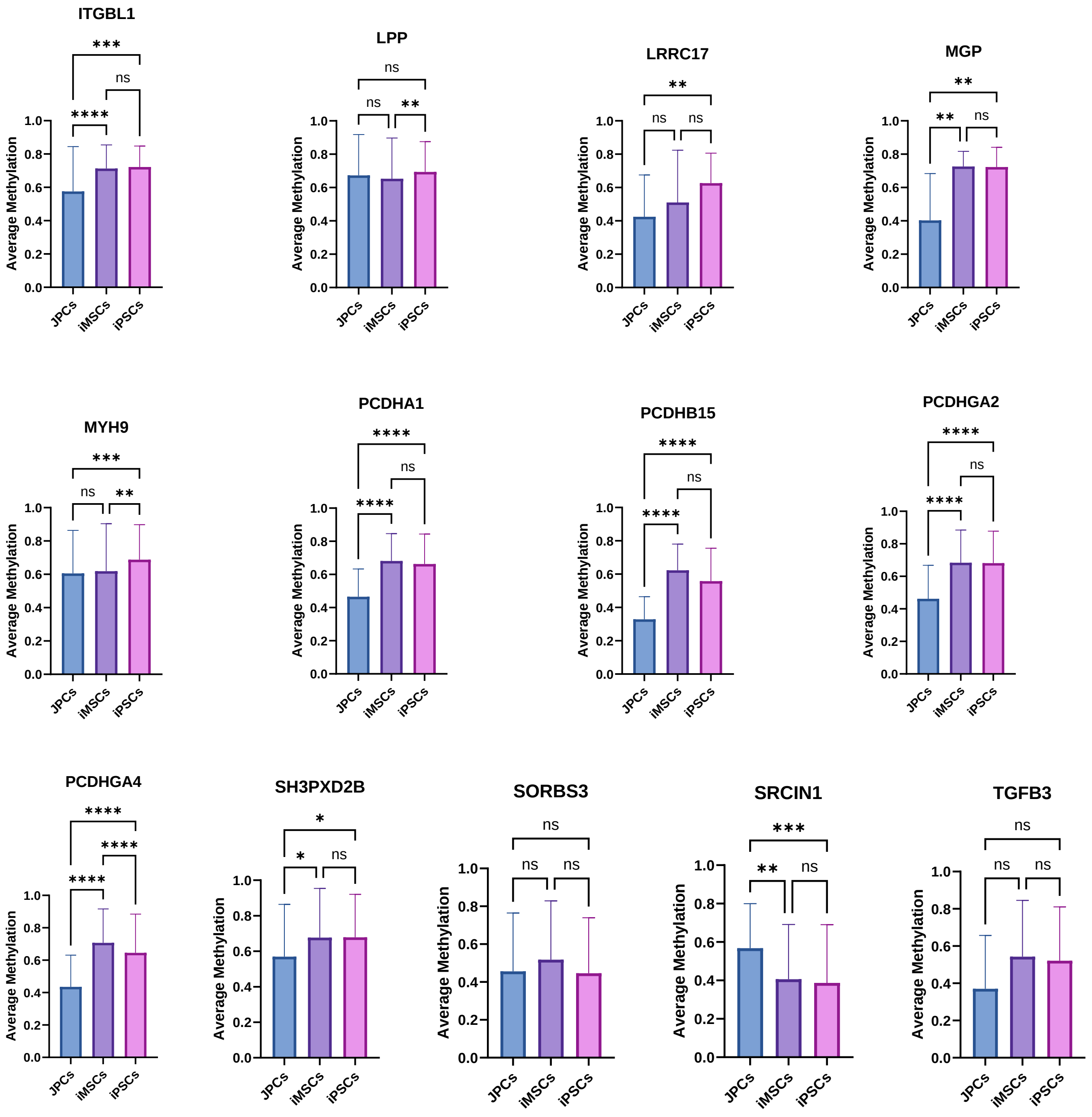

Supplementary Figure S2. Enriched genes contributing to GO terms "bone development", "cell adhesion", "extracellular matrix organization", "skeletal system development" and "skeletal system morphogenesis". Average methylation + SD of JPCs, iMSCs, and iPSCs is shown and the three groups were copared by repeated measures one-way ANOVA and Tuckey's multiple comparison test (* $p < 0.05$, ** $p < 0.01$, *** $p < 0.0001$, ****$p < 0.0001$).

Table S1. Age prediction (years) using listed methylation-based clocks (trained for different tissues) compared to the chronological age.

	Sex	Age	Group	Horvath, pan-tissue	Horvath2, skin and blood	BLUP, blood and saliva	EN, blood and saliva	Hannum, blood	Levine, blood	PedBE, buccal	Wu
Donor 1	M	21	JPC #1	33.31	35.74	38.16	41.76	11.59	12.17	6.25	5.84
			iMSC #2	-0.07	-0.69	-39.79	-39.33	6.38	-32.78	0.16	4.06
			iPSC #2	-0.35	-0.79	-34.06	-51.59	-6.83	-43.16	0.07	3.97
Donor 2	M	20	JPC #2	22.38	27.30	22.37	25.19	4.16	14.05	5.44	4.36
			iMSC #1	-0.06	-0.73	-38.58	-42.63	3.41	-28.45	0.12	4.67
			iPSC #1	-0.42	-0.81	-37.23	-55.75	-10.67	-50.41	0.06	4.21
Donor 3	M	30	JPC #3	31.94	40.39	38.27	44.52	8.51	9.92	5.60	6.64
			iMSC #3	0.12	-0.71	-36.64	-39.53	1.84	-28.85	0.17	3.60
			iPSC #3	1.56	-0.83	-34.95	-48.34	-12.07	-48.72	-0.07	4.43

Table S2. List of involved pathways extracted from the top 500 differentially expressed genes in JPCs versus iMSCs. Thereby, only the first four (marked in grey) listed pathways: complement activation (metaq = 0.009013), complement and coagulation cascades (metaq = 0.014027), glutathione metabolism (metaq = 0.044512) and prostaglandin synthesis and regulation (metaq = 0.015677), reached significant values and were shown to be elevated in JPCs compared to iMSCs.

Path-way.id	Pathway	logFC	meta,q
WP545	PATHWAY_WIKI:Complement activation_WikiPathways_20230410_WP545_Homo sapiens	1,908391	0,009013
WP558	PATHWAY_WIKI:Complement and coagulation cascades_WikiPathways_20230410_ WP558_Homo sapiens	1,162594	0,014027
WP100	PATHWAY_WIKI:Glutathione metabolism_WikiPathways_20230410_WP100_Homo sapiens	1,011135	0,044512
WP98	PATHWAY_WIKI:Prostaglandin synthesis and regulation_WikiPathways_20230410_ WP98_Homo sapiens	0,835223	0,015677
WP247	PATHWAY_WIKI:Small ligand GPCRs_WikiPathways_20230410_WP247_Homo sapiens	0,745045	0,523218
WP4719	PATHWAY_WIKI:Eicosanoid metabolism via cyclooxygenases (COX)_WikiPathways_ 20230410_WP4719_Homo sapiens	0,672073	1
WP167	PATHWAY_WIKI:Eicosanoid synthesis_WikiPathways_20230410_WP167_Homo sapiens	0,607029	0,612009
WP5052	PATHWAY_WIKI:Nephrogenesis_WikiPathways_20230410_WP5052_Homo sapiens	0,596456	0,148705
WP5176	PATHWAY_WIKI:Disorders of bile acid synthesis and biliary transport_WikiPathways_20230410_WP5176_Homo sapiens	0,595785	0,132338
WP5113	PATHWAY_WIKI:Antiviral and anti-inflammatory effects of Nrf2 on SARS-CoV-2 pathway_WikiPathways_20230410_WP5113_Homo sapiens	0,584363	0,1447
WP4969	PATHWAY_WIKI:RAS and bradykinin pathways in COVID-19_WikiPathways_20230410_WP4969_Homo sapiens	0,576686	0,346709
WP408	PATHWAY_WIKI:Oxidative stress response_WikiPathways_20230410_WP408_Homo sapiens	0,56721	0,038145
WP4815	PATHWAY_WIKI:Glycosaminoglycan degradation_WikiPathways_20230410_WP4815_ Homo sapiens	0,533642	0,114936
WP3599	PATHWAY_WIKI:Transcription factor regulation in adipogenesis_WikiPathways_ 20230410_WP3599_Homo sapiens	0,531619	0,203866
WP4705	PATHWAY_WIKI:Pathways of nucleic acid metabolism and innate immune sensing_WikiPathways_20230410_WP4705_Homo sapiens	0,511795	1
WP5276	PATHWAY_WIKI:Estrogen metabolism_WikiPathways_20230410_WP5276_Homo sapiens	0,501222	0,605306
WP4204	PATHWAY_WIKI:Tumor suppressor activity of SMARCB1_WikiPathways_ 20230410_WP4204_Homo sapiens	-0,49627	0,194295
WP5238	PATHWAY_WIKI:Cholestasis_WikiPathways_20230410_WP5238_Homo sapiens	0,492628	0,148705
WP4197	PATHWAY_WIKI:Immune response to tuberculosis_WikiPathways_20230410_WP4197_ Homo sapiens	0,491647	0,240795
WP4483	PATHWAY_WIKI:Relationship between inflammation, COX-2 and EGFR_ WikiPathways_20230410_WP4483_Homo sapiens	0,484453	0,194836
WP5101	PATHWAY_WIKI:Congenital generalized lipodystrophy_WikiPathways_20230410_ WP5101_Homo sapiens	0,471693	0,467959
WP205	PATHWAY_WIKI:IL-7 signaling pathway_WikiPathways_20230410_WP205_Homo sapiens	0,465333	0,071479
WP4721	PATHWAY_WIKI:Eicosanoid metabolism via lipooxygenases (LOX)_WikiPathways_ 20230410_WP4721_Homo sapiens	0,4531	0,32578
WP272	PATHWAY_WIKI:Blood clotting cascade_WikiPathways_20230410_WP272_Homo sapiens	0,428712	1

WP129	PATHWAY_WIKI:Matrix metalloproteinases_WikiPathways_20230410_WP129_Homo sapiens	0,425251	0,717985
WP364	PATHWAY_WIKI:IL6 signaling pathway_WikiPathways_20230410_WP364_Homo sapiens	0,421336	0,347635
WP4961	PATHWAY_WIKI:STING pathway in Kawasaki-like disease and COVID-19_WikiPathways_20230410_WP4961_Homo sapiens	0,414896	0,495132
WP3286	PATHWAY_WIKI:Copper homeostasis_WikiPathways_20230410_WP3286_Homo sapiens	0,414311	0,1755
WP5117	PATHWAY_WIKI:Cohesin complex - Cornelia de Lange syndrome_WikiPathways_20230410_WP5117_Homo sapiens	-0,41255	1
WP5088	PATHWAY_WIKI:Prostaglandin signaling_WikiPathways_20230410_WP5088_Homo sapiens	0,408995	0,931533
